# Editorial: mHealth for Non-Communicable Diseases

**DOI:** 10.3389/fpubh.2022.918982

**Published:** 2022-07-19

**Authors:** Ching-Hsien Hsu, Amir Alavi, Mianxiong Dong

**Affiliations:** ^1^Department of Computer Science and Information Engineering, Asia University, Taichung, Taiwan; ^2^Department of Medical Research, China Medical University Hospital, China Medical University, Taichung, Taiwan; ^3^Guangdong-Hong Kong-Macao Joint Laboratory for Intelligent Micro-Nano Optoelectronic Technology, School of Mathematics and Big Data, Foshan University, Foshan, China; ^4^Department of Civil and Environmental Engineering, Michigan State University, East Lansing, MI, United States; ^5^Department of Sciences and Informatics, Muroran Institute of Technology, Muroran, Japan

**Keywords:** e-health, healthcare, telemedicine, mobile health application, social medial

## Introduction

A Non-Communicable Disease is a non-infectious health condition that can last for a long period but cannot spread from one person to another. Cardiovascular Diseases, Diabetes, Cancer, and other Chronic Respiratory Diseases are categorized as Non-Communicable Diseases. In recent years, these diseases have become a great epidemic of our times, due to the increase of aging populations, changes in lifestyles, urbanization, and globalization. To overcome these epidemic situations in Non-Communicable Diseases, it requires a huge determination by adopting a continuously healthy lifestyle to prevent it. To mitigate this terrible epidemic, productive lives should be continuously monitored with healthy public guidelines and lifestyles. These unique challenges should be addressed more efficiently with more cost-effective techniques and also with the assistance of widely acceptable technology.

Mobile Health (mHealth) is a practice carried with the support of smartphones, patient monitoring applications, and other wireless devices for various medical and public health conditions. The face of health service delivery across the globe can be transformed by the use of smartphones and smart wireless technologies. Moreover, Mobile Health is the next wave of technology that will create a positive reaction among the patients, where it not only provides information about patient-reported outcomes but also helps in tracking a patient's behavior, symptoms, and what they're experiencing on a real-time basis. This can help in monitoring our lifestyle continuously by maintaining a healthy diet and assisting patients in managing any Non-Communicable Disease. This will also help clinicians to track patient symptoms and improve their experience. However, challenges related to the sharing of sensitive information of patients makes this effective platform risky in certain situations. Constituting effective guidelines and policies by governmental organizations regarding digital intervention shall enhance the future of mobile-based healthcare services. This special issue on “The Utilization of mHealth for Non-Communicable Diseases” invites research papers addressing the importance of mHealth and any of its various applications in the prevention, monitoring, and management of any Non-Communicable Disease.

This special issue aims at presenting the current state-of-the-art research and future trends on various aspects of mobile computing techniques for healthcare applications and attempts to build highly adaptive smart environments that can automatically adapt behaviors to the amount of available resources. The main areas covered by this special issue or main topics cover methodologies, modeling, analysis and newly introduced applications. Besides the latest research achievements, this special issue also deals with innovative commercial management systems, innovative commercial applications of Mobile Computing technology, and experience in applying recent research advances to real-world problem.

Papers selected for this special issue represent recent progress in the field, including works on mobile computing technologies, cloud based social computing, information systems, mobile social networks, and machine learning. All of these papers not only provide novel ideas and state-of-the-art techniques in the field, but also stimulate future research in the sustainable healthcare environment.

## Theory, Tools and Technologies for Mobile Health Applications

The perspectives of healthcare professionals (HCPs) are pivotal to co-development of self-management strategies for patients with diabetes. The paper by Yoon et al. entitled “*Healthcare Professionals' Views of Factors Influencing Diabetes Self-Management and the Utility of a mHealth Application and Its Features to Support Self-Care*” aims to explore factors influencing diabetes self-management in adult patients with diabetes from the perspectives of HCPs and their views of the value of mHealth application for diabetes self-management. This study identified a set of factors influencing self-management in adult patients with diabetes and useful app features that can empower patients to manage their conditions. Findings inform the development of a mHealth application, and its features designed to improve self-care.

The paper by Fruhwirth et al. entitled “*Evaluation of a Newly Developed Smartphone App for Risk Factor Management in Young Ischemic Stroke Patients: A Pilot Study*,” assessed the effect of a newly developed smartphone app for risk factor management in such a cohort. The app conveys key facts about stroke, provides motivational support for a healthy lifestyle and a reminder function for medication intake and blood pressure measurement. Specifically designed app interventions can be an easily to implement and cost-efficient approach to promote a healthier lifestyle in younger stroke patients. In this longitudinal pilot study, the authors proposed an efficient treatment of modifiable vascular risk factors decreases reoccurrence of ischemic stroke, which is of uttermost importance in younger patients.

The paper by Lim et al. entitled “*A Smartphone App-Based Lifestyle Change Program for Prediabetes (D'LITE Study) in a Multiethnic Asian Population: A Randomized Controlled Trial*,” aimed to assess whether a smartphone app-based lifestyle intervention programme would lead to weight loss, normoglycemia and improved metabolic indices in a multiethnic Asian population with prediabetes. An app-based lifestyle programme led to clinically significant weight loss and improved glycemia, and can potentially augment current standard care in the prevention of diabetes among an Asian multiethnic population.

## Social Medial and Health Management

Earthquakes inevitably affect the mental health of local residents. In seismically active regions of Southwest China, local rural residents' dilapidated housing with poor seismic performance aggravates the impacts of earthquakes on their mental health. To provide a foundation for applying telemedicine to assess the risk of mental health problems that rural residents in seismically active regions experience, the paper by Pan et al. entitled “*Telemedicine Assessment for the Mental Health of Rural Residents Based on the Safety Degree of Housing in Seismically Active Regions*,” studied whether the degree of safety of housing can affect mental health. In this study, nine villages near the epicenter of the 2019 6.0-magnitude earthquake in Changning County, China were randomly selected, and 162 valid questionnaires were completed. SPSS statistical software was used to analyze the collected data. The telemedicine assessment approach is expected to be used in the future for mental health evaluation and the large-scale data scoring of rural residents.

The scarcity of medical resources is a fundamental problem worldwide, and the development of information technology and the Internet has given birth to online health care, which has alleviated this problem. The paper by Yuchao et al. entitled “*Health Privacy Information Self-Disclosure in Online Health Community*,” constructed a model of influencing factors of self-disclosure intention about health privacy information in virtual health community. This study used the SPSS20.0 and AMOS21.0 to conduct exploratory factor analysis, confirmatory factor analysis, scale reliability and validity analysis, and the structural equation model to test the theory model. The conclusions of this paper will help supplement privacy calculus theory and expand the application scope of the attention based view. The proposed strategy can be used to stimulate the information contribution behavior of users in virtual health community and improve the medical service capabilities of online health community.

The paper by Alanzi et al. entitled “*Perception of Healthcare Providers About the Use of Social Media to Manage a Healthy Diet in Saudi Arabia*,” investigated the perception of healthcare providers about the use of social media to manage a healthy diet in Saudi Arabia. A cross-sectional study was designed to investigate the use of social media to manage a healthy diet. According to the perception of health providers, social media can be used to manage a healthy diet in Saudi Arabia. Also, the growing use of social media in this country represents the potential to create programs that encourage and promote healthy eating habits in the Kingdom. Instagram, YouTube, Snapchat and WhatsApp platforms can be used for this purpose.

## Conclusions

All of the above papers address either technical issues in mobile health technologies or medical information system or propose novel application models in the various mHealth management. They also trigger further related research and technology improvements in application of healthcare. Honorably, this special issue serves as a land-mark source for education, information, and reference to professors, researchers, and graduate students interested in updating their knowledge of mobile healthcare, medical of things, and novel application models for future medical information services and systems.

The special issue of this journal covers different aspects of the problem, from both the theoretical and the practical side. After a large open call, an international editorial committee selected twelve research papers. Each paper was reviewed by at least 3 reviewers.

## Author Contributions

All authors listed have made a substantial, direct, and intellectual contribution to the work and approved it for publication.

## Conflict of Interest

The authors declare that the research was conducted in the absence of any commercial or financial relationships that could be construed as a potential conflict of interest.

## Publisher's Note

All claims expressed in this article are solely those of the authors and do not necessarily represent those of their affiliated organizations, or those of the publisher, the editors and the reviewers. Any product that may be evaluated in this article, or claim that may be made by its manufacturer, is not guaranteed or endorsed by the publisher.

